# The role of the practice facilitators in Ontario primary healthcare quality improvement

**DOI:** 10.1186/s12875-015-0298-6

**Published:** 2015-07-30

**Authors:** Jyoti Kotecha, Han Han, Michael Green, Grant Russell, Mary I Martin, Richard Birtwhistle

**Affiliations:** Department of Family Medicine, Centre for Studies in Primary Care, School of Medicine, Queen’s University, 220 Bagot Street, P.O. Bag 8888, Kingston, Ontario K7L 5E9 Canada; Southern Academic Primary Care Research Unit, School of Primary HealthCare, Monash University, Building 1, 270 Ferntree Gully Road, Notting Hill, VIC 3168 Australia

**Keywords:** Practice facilitator, Quality improvement, Primary healthcare, Qualitative method

## Abstract

**Background:**

Practice facilitation is a key component of quality improvement in primary healthcare. Studies have reported the effectiveness of practice facilitation in improving quality management and care delivery. However, little has been published about practice facilitators’ training, facilitation activities, and their perceived role in quality improvement in primary healthcare. This study examined practice facilitators’ training and the perceptions of the practice facilitator role in a provincial primary healthcare learning collaborative quality improvement initiative in Ontario, Canada.

**Method:**

Descriptive and qualitative methods were used to outline the practice facilitator training as well as to look into the experiences and perceptions of practice facilitators and primary healthcare teams regarding the practice facilitation role in quality improvement. Data collection included training artifacts, activity logs, self-reflection reports, and semi-structured interviews with practice facilitators and primary healthcare participants. Reflections and interviews were analyzed to identify the role of the practice facilitators from their own experience, and from the perspective of the participants. Descriptive statistics were used to learn about categories of facilitation activities undertaken and frequency of these activities.

**Results:**

Sixteen practice facilitators and seven family healthcare teams participated in the study. Practice facilitators received a two-day intensive training workshop and continued training. Their time was spent mostly working directly with participating teams, continued learning and training, communications and administration. They served as coaches, resource providers, enablers and motivators. Participating teams expressed satisfaction with the practice facilitator role, although they had hoped this position would provide onsite and hands-on support in conducting activities of quality improvement at the practice level.

**Conclusions:**

Practice facilitators played a crucial role in the implementation of quality improvement in Ontario’s learning collaborative program. The practice facilitator role is perceived to be that of a coach, enabler and motivator. This study suggests that the practice facilitator successfully supported participating teams to undertake quality improvement activities in primary healthcare settings.

## Background

Over the last decade the province of Ontario in Canada has introduced significant reforms to the organization and delivery of primary healthcare [1]. One of these changes was the establishment of inter-professional primary healthcare teams (PHTs) known in Ontario as family health teams, and engagement of PHTs in quality improvement (QI) initiatives. The Quality Improvement and Innovation Partnership (QIIP), now combined into Health Quality Ontario (HQO), was a key partner in moving the QI agenda forward in PHTs [2]. The role of the QIIP was to support PHTs to develop strong inter-professional care teams and build capacity to implement QI programs within their practices. During 2008 and 2010, the QIIP launched three waves of Learning Collaboratives (LC) to train PHTs on methods of QI with a focus on improvement in chronic disease management, illness prevention, access to care and office redesign. The QIIP LC program design was based on the Institute for Healthcare Improvement (IHI) Breakthrough Series Model [3, 4]. The IHI model is a structured adult learning method for facilitating quality improvement in healthcare services. Each wave of the QIIP LC was approximately 15 months in length, consisting of three separate two-day learning sessions, action periods between learning sessions, and a summative congress at the end of the program. To facilitate PHT participation and engagement in the LC, QIIP used external practice facilitation to support team development and application of QI knowledge into practice.

Practice facilitation has been suggested to be a key “component of quality management” [5] or a “QI process” [6]. The practice facilitation model was introduced to primary care in England as early as the 1980s [7, 8], and then was adopted in the United States and Australia [5, 9–14]. In Canada one of the first documented primary healthcare facilitation studies was conducted in 1997 in Ontario by Lemelin et al., who reported substantial improvement in the delivery of preventive services in the study arm supported by a practice facilitator [15]. However, only recently has the practice facilitator role been used in QI initiative programs in Canada [6, 16], and it is still used infrequently [17].

Practice facilitators, also known as practice coaches or QI coaches, work closely with practices to identify areas of improvement, set improvement goals, provide tools, and facilitate QI activities and practice redesign in primary healthcare settings [17]. They are described as healthcare professionals who assist practice staff to assess care processes, plan implementation measures and improve strategies for illness prevention [5, 6]. They also play the role of change agents who train practices to understand and use data effectively to drive QI, promote an interdisciplinary team approach to care, and increase capacity for creating and maintaining QI infrastructure within practices [18].

Practice facilitation in primary healthcare has been shown to have a positive effect on adoption of evidence-based delivery of prevention [19] and chronic illness care [20]. However these studies often provide little detail regarding practice facilitator’s training, number of practices supported, and intensity and duration of practice facilitation or specifics about activities they perform [17]. Thus, despite external facilitation being shown to have positive effects [6, 18], little has been published about practice facilitators’ actual activities and perceptions of their role from their own experience and from that of primary healthcare practices who received their support. The purpose of this study is to describe the training received by practice facilitators in the QIIP LC program in Ontario, Canada, and the role of these practice facilitators as perceived through the lived experiences of the facilitators themselves and by the participating PHTs.

## Method

We conducted a descriptive and qualitative study. A phenomenological approach was used for the qualitative study, as this approach supports the exploration of how participants make sense of experiences both at the individual level and as a shared experience. Semi-structured interviews were conducted to explore the lived experience [21] of the practice facilitators and the participants regarding practice facilitators’ role in the QI program, and the knowledge they shared from their experiences [21, 22].

### Sampling and recruitment of participants

We aimed to recruit all 16 practice facilitators who were part of the QIIL LC program to participate in recoding activities, providing monthly reflection notes, and the interviews. Recruitment of the practice facilitators was through a study information session given to them during a regular practice facilitator teleconference. All 16 consented to participate in the recoding of activities and in providing monthly reflection reports. One facilitator agreed to support piloting the interview script and 15 consented to participate in the interviews.

A purposeful sampling strategy was used to select participating PHTs for interviews. A sample of 8 PHTs were identified to reflect a maximum variation of the participating teams in terms of settings (43 % rural and 57 % urban), practice size (57 % >5 physicians), and geographical regions across Ontario (71 % southern and 29 % northern). Practice facilitators supported the recruitment of the PHT interview participants through distribution of study information and connecting interested participants to the research team. Key informants from seven out of the 8 PHTs consented to participate in the study. Typically, key informants from these practices were the team leaders, executive directors or clinical managers. The final sample of seven PHTs was determined from the ongoing analysis of the interviews and ceased once saturation of themes was achieved and no new ideas and concepts were introduced.

### Data collection

Artifacts related to practice facilitator recruitment and training were collected from the QIIP website and administrative documents. These included: job postings, job description, training material, training schedules, and documents about practice facilitator competency requirements.

An electronic logbook was designed to capture practice facilitators’ demographic information, daily activities and amount of time they spent on each noted activity. These logbooks were maintained by the practice facilitators on a daily basis and were submitted to our research team at the end of each month. Additionally, the practice facilitators were also required to submit a monthly self-reflection report on their ability to accomplish facilitation tasks.

Semi-structured telephone interviews (15–45 min) were conducted with 15 consenting practice facilitators and 7 consenting individuals from the PHTs who were recruited towards the end of their participation in a learning collaborative that lasted approximately 14–16 months. The interview scripts were piloted on a volunteer practice facilitator and PHT team participant who did not participate in this study. These pilot interviews allowed us to refine the interview script for clarity. The interview questions were focused on learning about the participants’ understanding of the practice facilitator role and how practice facilitators assisted PHTs to implement QI in clinical practice during the QIIP LC program. The interviews were audio taped and transcribed verbatim. Data saturation was achieved upon the completion of 7 interviews with individuals from the participating PHTs.

### Data analysis

All training artifacts collected were documented to reflect QIIP practice facilitator training and qualification requirement. The practice facilitators’ logbooks were analyzed to identify major daily activities and the distribution of their time to these major activity categories. Practice facilitators’ monthly reflections and interview transcripts were coded using NVivo software [23] and analyzed inductively for emerging themes through an iterative and interpretive approach [21]. Interviews of PHT participants were analyzed inductively and the themes were crosschecked with the themes from the practice facilitator interviews. Recurrent themes from both groups of interviews were clustered and organized into related concepts. The strategy of immersion and crystallization was used to analyze the data; the researchers (JK & HH) examined the texts and discussed topics and themes to provide a comprehensive view of the themes and overarching concepts [24]. The shared and collective experiences of the practice facilitators and PHT participants are reported in group views with data sources being specified as “Facilitator Reflection Reports”, “Facilitator Interviews” and “PHT Interviews”.

### Credibility and trustworthiness of data

Ethics approval was obtained for this study from the Health Science Research Ethics Board of Queen’s University. All interview transcripts and reflection reports were coded individually by two research associates who compared and discussed their coding on a regular basis. Analysis was team reviewed to minimize the personal bias of an individual researcher. Researchers were not involved in the implementation of the QIIP LC programs.

## Results

Analysis of the QIIP documentation and the practice facilitator activity logs provided descriptive information regarding: (a) practice facilitator skills and training, (b) practice facilitator scope of practice and (c) activity categories and time allocation to these activities. From the analysis of the monthly practice facilitator reflection notes and interviews, four overarching themes emerged: (a) work scope of the practice facilitators within the QIIP LC program, (b) role of the practice facilitator in healthcare practices to drive QI, (c) the needs and expectations of the PHTs, and (d) satisfaction with and expectation of the practice facilitator role in primary healthcare QI initiatives. It was apparent that there were strong similarities across the entire practice facilitators group, therefore, the shared experiences of the practice facilitators are reported as a common voice specified as “Facilitator Reflection Reports” and “Facilitator Interviews”. The PHT members, who also expressed similar ideas, are reported as a group specified as “PHT Interviews”.

### QIIP practice facilitator skills and training

The QIIP hired 16 practice facilitators in two cohorts, 12 facilitators in the first cohort and an additional four facilitators in the second cohort. The practice facilitators were part time (0.5-0.75 full-time-equivalent) employees that worked out of a home office. Each facilitator worked with teams assigned to them throughout their participation in the LC program, which was approximately 15 months in length. The practice facilitators had formal university degrees in different areas of study, including: nursing, social work, education and health policy. They also had work experience and skill sets related to: healthcare services, healthcare data management, health research, chart auditing, and electronic medical record (EMR) use.

The practice facilitators were provided with training to build their facilitation and primary healthcare delivery competency. This training was based on the following core QI competencies [25]:

- Clinical knowledge related to diabetes guidelines and delivery design,
- QI methodology, such as plan-do-study-act (PDSA) and process mapping,
- Facilitation skills for team development and practice coaching,
- Communication strategies such as conflict resolution and presentation skills, and
- Information management, including data analysis and use of EMR.

The QIIP felt that these competencies would enable the practice facilitators to fulfill seven roles of a change agent: QI expert, communicator, collaborator, system thinker, manager, educator and scholar, and leader.

Two weeks prior to the start of the first wave of LC, the first cohort of 12 practice facilitators attended a two-day intensive face-to-face workshop. Following the initial training workshops, additional supervision was provided to individual facilitators to meet their specific needs. Ongoing practice facilitator training continued throughout the LC program through additional face-to-face meetings, small group workshops, teleconferences, self study modules, and personal mentoring. For the second cohort of four practice facilitators, a “lessons-learned” training session was incorporated in which the first cohort of facilitators mentored their new colleagues, shared experiences and lessons learned.

### What did the practice facilitators do in the QIIP LC Program?

Practice facilitators were assigned to primary care teams based on proximity to their home offices. Because their workload and schedule changed day to day, it was hard to describe a typical day or week for the practice facilitator. “*I have to keep very flexible because things can change at the drop of the hat so there is a certain amount of flexibility to my day or week*” (Facilitator Interviews). However, even given such flexibility, four main categories of activities (Table 1) emerged from the data provided in their activity logs and reflection reports.

1. *Working with assigned primary healthcare teams.* The practice facilitators spent about a third of their work time with their teams virtually and face to face. Using a web-based virtual office, teleconferences, emails and phone calls, the practice facilitators facilitated team meetings, coached the teams to use implement change cycles (Plan-Do-Study-Act, PDSA) for planning and implementing QI activities, and mentored them to document and review changes and report on outcomes. They also traveled to practice sites to provide hands-on support. “*It’s much more effective to spend time with teams and on a one-to-one basis. It’s better in terms of helping meet individual learning/coaching needs and for building working relationships*” (Facilitator Reflection Reports).
2. *Administrative tasks*. The practice facilitators used over a quarter of their work time on administrative work. They searched for specific knowledge and strategies to address the challenges faced by the PHTs, sorted out questions and answers through emails, analyzed the best practice guidelines, and documented team progress “*One team has some unique challenges, for example, no EMR, limited physician resources. I offered to help connect them to two teams that had similar issues*” (Facilitator Interviews).
3. *Practice facilitator training and education.* About a quarter of the practice facilitators’ time was spent on receiving ongoing practice facilitation training and education during and between the learning sessions. Although the facilitators had related working experiences and competencies, they still needed to learn or update their knowledge and skills related to the QIIP LC program, including methodology for improvement, use of the PDSA tool, process mapping, methods for measuring indicators of health outcomes, organizational development, advance access, and coaching skills. “*I am really enjoying the online facilitation course and the webinars, the training plans are responsive to needs*” (Facilitator Interviews).
4. *Contact with the QIIP Team and colleagues.* The practice facilitators used about 13 % of their time communicating with the QIIP Administration Team and their colleagues. They reported the progress and issues of their teams in applying QI to practice and shared with each other the challenges and successes of working with the teams. “*Other PFs [practice facilitators] are a great support and resources*” (Facilitator Interviews). The communications within the practice facilitator circle and with the QIIP Administration Team enabled the individual facilitators to learn from each other and be better able to address the needs of the teams.

**Table 1:** Practice facilitator major activities and time distribution generated from activity logbooks

Practice facilitator major activities	Work time distribution
Working with assigned primary healthcare teams	34 %
Administrative tasks	27 %
Practice facilitator training and education	26 %
Contact with the QIIP Team and colleagues	13 %

### What role did the practice facilitators play in the PHTs’ drive to QI?

The practice facilitators fulfilled the responsibilities of coach, facilitator and supporter. In their reflection reports and interviews the practice facilitators also described themselves as a resource, guide, enabler and motivator, and this corresponded to the description provided of the role through the primary healthcare provider interviews.

#### Coach, facilitator and supporter.

The practice facilitators believed that their most important role was to support the teams and facilitate quality improvement tasks to be implemented into clinical practice. “*We are coaches, we’re coaching teams in quality improvement practices as it relates to this Collaborative and the specific focus of the Collaborative*” (Facilitator Interviews).

The PHTs agreed that the practice facilitators supported team functioning. One PHT member mentioned, *“They come in during team meetings to look at team dynamics, to see where the group is struggling and offer advice and guidance to how to move forward”* (PHT Interviews). The facilitators helped the teams to establish goals. Another PHT member stated, “*She [practice facilitator] helped us with our plan to scale it back to something that’s reasonable, helped us focus on really good planning and evaluation techniques around the PDSA stuff*” (PHT Interviews). When the teams had the challenge of where to start a change or how to achieve more improvement, their practice facilitators would facilitate decision making* “[The practice facilitator is] really drilling down to where we could get the easy wins, how we could start engaging more”* (PHT Interviews).

#### Accessible resource and guide.

Throughout the QIIP LC program the practice facilitator provided information resources to the teams, including learning materials, change tools, and experiences of other teams. “*I think providing teams with information and documents to help them move their work, so really being sort of an agent of kind of knowledge*” (Facilitator Interviews).

The PHTs were impressed by the practice facilitators’ knowledge. One PHT member noted, “*She [practice facilitator] was very knowledgeable not only as related to the actual process but also…lots of other tools related to process mapping*” (PHT Interviews). Although the teams attended the LC learning sessions and had access to the learning materials posted on the QIIP virtual office, they still needed the practice facilitators’ guidance and suggestions to specific problems encountered in practice. *“[practice facilitator is] very available, if I emailed her, she’d respond that same day, I could call her any time I had a question or an issue and she’d address it as soon as possible*” (PHT Interviews). The teams had trust in their practice facilitators who were *“able to lead us along the way and provide us with some insight and… tools to be able to complete the processes as best as possible”* (PHT Interviews).

#### Enabler and motivator.

In addition to supporting the teams through coaching, facilitation and guidance, the practice facilitators felt that they had to push the teams to move forward in the QIIP LC program. *“[I’m] an enabler or empowerment I guess, I really believe that part of my role is to make sure that teams are functional all on their own*” (Facilitator Interviews). The practice facilitators had to motivate the teams whose progress was set back due to challenges and busy practice. “*[I] definitely have a role of being a motivator, an encourager, someone to help them get back on track sometimes when they’ve gotten distracted by all the other things that go on in their practice*” (Facilitator Interviews).

The PHTs also felt that the practice facilitators enabled them to see themselves *“from the outside and look at the bigger picture”* and *“help us [teams] see the challenge and opportunity”* (PHT Interviews). The teams stated that they needed the motivation and encouragement from the practice facilitators from time to time. One PHT member stressed, *“You need that person, that link, you know, that positive beacon to keep you going and to keep you re-focused”* (PHT Interviews). Some teams even believed that without a practice facilitator, their participation in the LCs would have faltered*. “I think that the teams would really suffer if they didn’t have a facilitator to check in with and to keep in contact with”* (PHT Interviews).

### How did the practice facilitators meet the needs and expectations of PHTs?

Overall the PHTs felt that their practice facilitators met their expectations by supporting them through the activities of the QIIP LC program. “*She’s come out and given us some tools as far as … being able to accomplish and measure some of the things that we were looking. I think that’s what I expected and that’s what she did*” (PHT Interview).

On average each PF supported six different primary healthcare teams. As a result, practice facilitators and PHT participants felt that that the time to meet with each individual team was limited and that they needed more time to get to know each other, especially prior to jumping into the work of the QIIP LC program. The practice facilitators felt they needed more time to prepare themselves to step into the practice facilitator role. One facilitator noted in her reflection report, “*We had a really short window of time from the time we were hired until the start of the first learning collaborative, so perhaps a bit more time up front would have helped*” (Facilitator Reflection). It was imperative for the practice facilitators to learn how the teams really operated before they began their support role. Another facilitator stated:

*…[need for more time] understanding Family Health Teams[PHT] -- how they function, not just necessary the political, the money, the administrative side of it, but the day to day functioning of a Family Health Team and the differences between them and what that looks like, and the challenges they face.* (Facilitator Interviews).

The PHTs also stated that it would have been more helpful if they met their practice facilitators before the start of the program because it took time for the facilitators to get to know their teams. “*We didn’t meet our practice facilitator until the day we had our first learning session, and that meant that the individual had to spend a couple of months coming up to speed on our practice*” (PHT Interviews). PHTs could feel the struggling experienced by their practice facilitators at the beginning of the LC program. “*They were stepping into a role that hadn’t been defined yet, so I think that there was some confusion on their part”* (PHT Interviews). Not until after a period of time did the teams and the practice facilitators develop a comfortable working relationship that would then allow the practice facilitators to really support the teams. *“The comfort level and familiarity with each other … the more she (practice facilitator) knows about what’s been going on here, the more helpful she’s been”* (PHT Interviews).

#### Expectation of more on-site and hands-on support of the practice facilitators

Although PHTs understood the nature of the external practice facilitation model, they still hoped that the practice facilitators could have been more integrated into their practice allowing them to provide more hands-on support to actually conduct some of QI activities within the practice. *“It would have been nice to have these individuals … with us one day a week or actually really kind of part of the practice so that they became a face [for quality improvement]”* (PHT Interviews). The PHTs had this expectation because carrying out QI activities by themselves was challenging due to competing priorities and a heavy clinical workload.

## Discussion

Our study collected multiple types of data and used descriptive and qualitative data analysis to examine the practice facilitators who were trained to facilitate PHTs’ participation in the QIIP LC program. This study documented how the practice facilitators were trained and what they did to facilitate the PHT participation in the LC program. The results indicated that PHTs were satisfied with the practice facilitator role, commenting that they enabled and motivated them to implement QI programs. The QIIP practice facilitators’ training was a combination of intensive workshop learning and continual learning during the rollout of the LC and throughout the program. QIIP’s ongoing support, communication and sharing of experiences and lessons learned among the facilitators themselves, as well as learning from working with the teams, contributed immensely to practice facilitation training. The practice facilitators' related professional experiences enabled them to quickly acquire new knowledge pertaining quality improvement methods during their participation in a short, intensive training workshop. However, on-going education and sharing of lessons and experiences with peers were felt to be beneficial and better equipped them to address the needs of their teams. Our documentation of the QIIP practice facilitators’ training and their actual activities in the LC program can be a reference for other innovative healthcare programs that plan to use an external practice facilitation strategy.

The practice facilitator’s role has been described in literature by Taylor et al. [18] as trained individuals who help and enable practices or teams to undertake QI initiative, understand and use data for QI and develop capacity for continuous QI, and address the challenges of implementing evidence-based guidelines within the primary care settings. Knox et al. [26] stated that practice facilitators are professionals who coach, mentor or conduct activities that support healthcare teams to implement changes that lead to improved quality of care. The results of our study do not only confirm the role of practice facilitators as described in the literature, but also brings a deeper insight of the practice facilitation role through the lived experiences of the practice facilitators and PHTs. In addition to coaching PHTs the facilitators also served as recourse providers, guides, enablers and motivators to the PHTs. Participating PHTs felt that the practice facilitators were crucial to their ability to participate fully in the QIIP LC program and to initiate QI programs in their practices.

The external practice facilitation model has been widely adopted in implementation of QI or other change initiatives in Canada [7, 15, 27, 28], and its effectiveness has been reported [29]. Our study also indicates an overall satisfaction of the PHTs with the external practice facilitation services that they received during their participation in the QIIP LC program. Nevertheless, the interview data revealed inadequate provision of on-site and hands-on assistances in the QIIP LC external facilitation. The practice facilitation logbooks indicated that the facilitators had made full use of modern information technology, such as virtual office and telecommunication, to interact with the teams in this external practice facilitation approach. However, PHTs and the practice facilitators felt that face-to-face interaction and onsite hands-on support were still valuable and not replaceable. The PHTs in particular expressed the need for more face-to-face and hands-on assistance from practice facilitators to meet the unique challenges of carrying out QI activities in their practices. Our findings suggest that embedding more on-site and hands-on support into the external practice facilitation model may increase the effectiveness of practice facilitation programs. Our suggestions may have implications for implementation of external practice facilitation programs in the future, such as reducing the number of teams assigned to any one practice facilitator, or increasing work time to allow practice facilitators the ability to interact more frequently with each team. In the QIIP LC program the practice facilitators were part-time, employees. Thus, we acknowledge that our suggestion for increasing hours of work may increase program costs.

This study was limited in that it did not recruit the PHTs from the teams that that participated in the third wave of the LC program due to a limited timeframe. As well, it did not look into the impact of the practice facilitators on QI outcomes within the PHTs that participated in this study.

## Conclusion

This study documents practice facilitator training, what skills they required to support QI implementation in PHTs during the QIIP LC program and it details the activities that the practice facilitators were engaged in to fulfill their facilitation responsibilities. The lived experiences of both the practice facilitators and the PHTs described how the role of practice facilitators met the needs and expectations of the PHTs. During the QIIP LC program, the practice facilitators enabled PHTs to initiate QI change, facilitated application of knowledge and QI tools to improve clinical practice, served as information resources and links, and motivated the teams to engage in QI activities. Limitations of the external practice facilitation model were obvious interms of insufficient onsite and hands-on support in actually supporting QI activities at the practice level. Although this study did not assess the effectiveness of QIIP practice facilitation on QI outcomes in primary care, the results reported provides references for practice facilitator training programs and suggests that the external practice facilitator role can enable quality improvement in primary healthcare settings.

## Abbreviations

EMR: Electronic medical record
PHTs: Primary healthcare teams
HQO: Health Quality Ontario
LC: Learning collaborative
PDSA: Plan-do-study-act
QI: Quality improvement
QIIP: Quality Improvement and Innovation Partnership
